# Ultra-Processed Foods and Nutritional Dietary Profile: A Meta-Analysis of Nationally Representative Samples

**DOI:** 10.3390/nu13103390

**Published:** 2021-09-27

**Authors:** Daniela Martini, Justyna Godos, Marialaura Bonaccio, Paola Vitaglione, Giuseppe Grosso

**Affiliations:** 1 Department of Food, Environmental, and Nutritional Sciences, Università degli Studi di Milano, 20133 Milan, Italy; daniela.martini@unimi.it; 2 Department of Biomedical and Biotechnological Sciences, University of Catania, 95123 Catania, Italy; giuseppe.grosso@unict.it; 3 Department of Epidemiology and Prevention, IRCCS NEUROMED, 86077 Pozzilli, Italy; marialaura.bonaccio@moli-sani.org; 4 Department of Agricultural Sciences, University of Naples Federico II, 80055 Portici, Italy; paola.vitaglione@unina.it

**Keywords:** ultra-processed food, diet quality, sugar-sweetened beverages, sweets, nutrients, nationally representative

## Abstract

Excessive consumption of ultra-processed foods (UPFs), as described by the NOVA classification system, represents a potential threat to human health. The nutritional composition of UPFs may explain their observed adverse effects. The present study aimed to provide a quantitative meta-analysis of nationally representative surveys on the consumption of UPFs and the dietary/nutrient composition of respondents’ diets. A systematic search for relevant studies published prior to July 2021 was conducted via electronic databases. The studies that provided the dietary/nutrient composition of foods categorized according to the NOVA classification system were selected. The association between UPFs and other dietary variables was modelled using ordinary least squares linear regression based on aggregated data extracted from the selected articles. Consumption of UPFs represented up to 80% of total caloric intake in the US and Canada, with confectionery and sugar-sweetened beverages being the most consumed items. When considered in relation to other food groups, an inverse linear relation between UPFs and less-processed foods was evident. Increased UPF intake correlated with an increase in free sugars, total fats, and saturated fats, as well as a decrease in fiber, protein, potassium, zinc, and magnesium, and vitamins A, C, D, E, B12, and niacin. In conclusion, the data indicate that increased UPF consumption negatively affects the nutritional quality of diets.

## 1. Introduction

The current scientific evidence indicates that a large share of non-communicable diseases is influenced by dietary risk factors [1,2]. While several studies provide evidence for the health benefits associated with the appropriate consumption of plant-derived foods—such as fruits and vegetables [3], whole grains [4,5], and nuts and legumes [6,7]—a growing body of literature suggests that, in addition to their nutrient content, the production and formulation methods of foods may also play a role in their effect on human health. With the rise in concern regarding food processing [8], major interest has been paid to ultra-processed foods (UPFs), defined by the NOVA food classification system as industrially manufactured products containing little to no whole foods and characterized by cosmetic alterations and additives that increase their palatability and sensorial properties [9]. In addition to these formulation features, most products that are classified as UPFs are highly palatable, convenient, and easily available [10]. A recent analysis of data from the last 15 years revealed that sales of UPFs are markedly higher in North America, Western Europe, and Australasia than in other regions; however, substantial growth in Latin America, Eastern Europe, North Africa, the Middle East, and Central and Eastern Asia was noted and is projected to continue over the coming years [11]. The rise in consumption of UPFs is associated with several aspects of modern lifestyles, including longer eating windows, frequent snacking, eating outside the home, and the ease of access and economic affordability of UPFs [12]. Moreover, UPF supply across the globe relies on emergent (in developing countries) or consolidated markets (in developed ones), with their availability increasing with the accessibility of supermarkets and fast-food chains [13].

Most studies on UPFs in relation to human health report a substantial association with an increased risk of obesity [14]; however, recent evidence underlined that the potential health risks associated with their consumption may also include a higher risk of adverse cardio-metabolic outcomes (including cardiovascular diseases, hypertension, and metabolic syndrome) [15], depression, irritable bowel syndrome, adolescent asthma and wheezing, frailty, and overall mortality [16,17]. In addition to products containing additives and preservatives, UPFs—as classified by the NOVA system—may also include energy-dense products and those high in added sugars, fats, or sodium, which may explain, from a mechanistic point of view, their observed detrimental effects on human health [18]. However, occasional consumption of unhealthy foods or UPFs, in terms of net amount or as a percent of total energy intake, is unlikely sufficient to exert such negative effects [19].

The majority of existing studies that assess UPF consumption at the national level are conducted based on sales; as such, a comprehensive and combined evaluation of individuals’ data regarding UPF intake and diet nutritional quality is lacking. Thus, it is important to understand how the consumption of UPFs correlates with the overall nutritional profile and quality of diets. In this study, we aimed to systematically review the existing data from nationally representative surveys regarding UPF consumption in relation to dietary nutritional profiles, and to perform a meta-analysis of the results in order to understand the similarities or discrepancies that exist between countries.

## 2. Materials and Methods

We followed the Preferred Reporting Items for Systematic Reviews and Meta-Analyses (PRISMA) guidelines—an evidence-based minimum set of items for reporting in systematic reviews and meta-analyses—when providing information regarding the work conducted in this study (Supplementary Table S1).

### 2.1. Study Selection

Identification of all articles published up to July 2021 was conducted via a systematic search of MEDLINE and EMBASE. The search strategy was based on the combination of the following key terms: “ultra-processed foods”, “nutrient”, “nutritional profile”, “diet”, and their synonyms. The references cited in each of the retrieved articles selected for review were also considered. Studies were considered eligible if they (i) provided classification of the processing level using the NOVA classification system; (ii) provided the content of specific dietary components for at least three categories of UPF consumption based on contribution to total energy consumption; and (iii) were written in English. All studies based on NOVA classification should allocate each food item into the following groups: (i) unprocessed or minimally processed foods; (ii) processed culinary ingredients; (iii) processed foods; and (iv) ultra-processed foods. Study quality was assessed by the Strengthening the Reporting of Observational studies in Epidemiology (STROBE) checklist, which is used for cohort studies exploring various domains described across the studies included in a systematic review (such as background/rationale, objective, study design, setting, participants, variables, data sources, bias, study size, quantitative variables, statistical methods, outcomes and main results, limitations, interpretation, and funding) [20].

### 2.2. Data Extraction

Data extracted for each category of exposure included (i) the number of individuals; (ii) the mean energy intake of NOVA food groups and subgroups across quintiles of dietary share of UPFs; (iii) mean dietary content of macronutrients as a percentage of total energy (%E) across quintiles of energy contribution of UPFs; and (iv) the mean density of micronutrients (in grams per 1000 kcal or milligrams per 1000 kcal, etc.) across quintiles of energy contribution of UPFs. Study selection and data extraction were performed by two researchers (J.G. and D.M.) and any discrepancies were resolved by discussion until consensus was achieved.

### 2.3. Statistical Analysis

A two-stage approach was applied to the statistical analysis. In the first step, the association between UPF and each specific food item’s contribution to total energy intake was modelled using ordinary least squares linear regression using the data from individual studies. We used data aggregated at the group level: participants were divided into quintiles according to the energy contribution of UPFs to their typical diet. We extracted from articles the mean values for dietary share of UPFs, and the mean dietary content of macronutrients expressed as %E. Both the intercept and slope of the dose–response relationship were retrieved. Next, for each food item, a separate bivariate meta-analysis with weights proportional to the study sample sizes was performed to simultaneously synthesize slopes and intercepts [21]. Unstructured variance-covariance matrices and random effects were incorporated into the models. The slopes reported in the tables represent the variation in intake of a specific food item when the share of %E from UPFs increased by 1%. In addition to presenting the value of the slope coefficient, to better reflect the relationship between UPFs and the other dietary factors investigated, we estimated the dietary share of these factors at three arbitrarily chosen points corresponding to 15, 50, and 75% of total energy intake from UPFs, intended to represent their low, medium, and high consumption, respectively. A meta-analysis of Pearson correlation coefficients was performed to evaluate the strength of the association between individual products and ingredients in diets and the contribution of UPFs to total energy intake. All analyses were performed with R software version 4.0.2 (Development Core Team, Vienna, Austria).

## 3. Results

### 3.1. Study Selection

**Figure 1:**
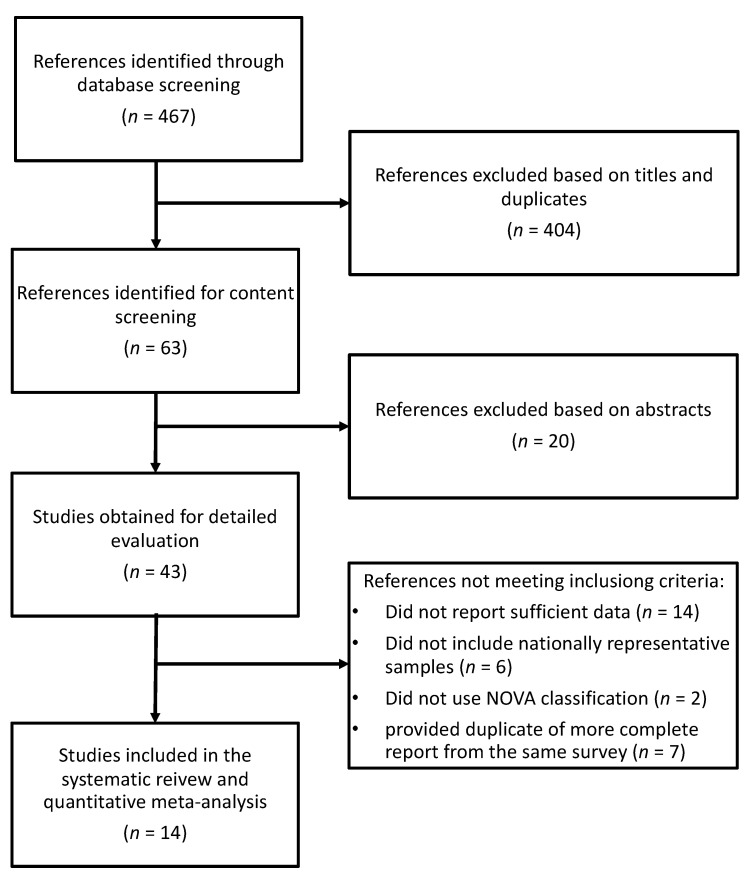
Screening and selection process for study articles exploring the distribution of foods and nutrients by quantiles of ultra-processed food intake (as a percentage of daily energy).

The search strategy identified 467 studies to be screened, of which 43 were selected for full-text evaluation after exclusion based on titles and abstracts (Figure 1). A total of 29 additional studies were excluded for the following reasons: (i) did not report sufficient data (*n* = 14); (ii) did not include nationally representative samples (*n* = 6); (iii) did not use the NOVA classification system (*n* = 2); and (iv) were duplicates of previously selected more complete reports from the same survey (*n* = 7). Therefore, a total of 14 studies providing data for 13 unambiguously nationally representative samples were included in the present meta-analysis [22,23,24,25,26,27,28,29,30,31,32,33,34,35].

### 3.2. Study Characteristics and UPF Consumption

All studies included were compliant with the STROBE statement. The main characteristics of the cohorts included are presented in Table 1. Four studies were conducted in South American countries (including Mexico, Brazil, Chile, and Colombia), five in Europe (including the UK, Portugal, France, and Italy), two in North American countries (including the US and Canada), one in Taiwan, one in Korea, and one in Australia. The sample sizes ranged from around 2000 to nearly 40,000 individuals, with children and adults included in all the studies except for three that recorded data only for older adolescents [26] and adults [32,33,34]. Seven studies assessed dietary intakes through a single 24-h recall, five studies used two 24-h recalls, and one study used a 4-day diary.

The average consumption of UPFs as %E varied greatly across the studies, ranging from 15.9, 19.5, and 21.5%E in Colombia, Taiwan, and Brazil, respectively, to 47.7, 56.8, and 57.5%E in Canada, the UK, and the US, respectively. Importantly, the mean consumption of UPFs in the highest quintile groups of the three latter countries reached 76.2, 78.1, and 80.7%E, respectively (Table 1).

### 3.3. Correlation with Dietary and Nutritional Factors

**Figure 2:**
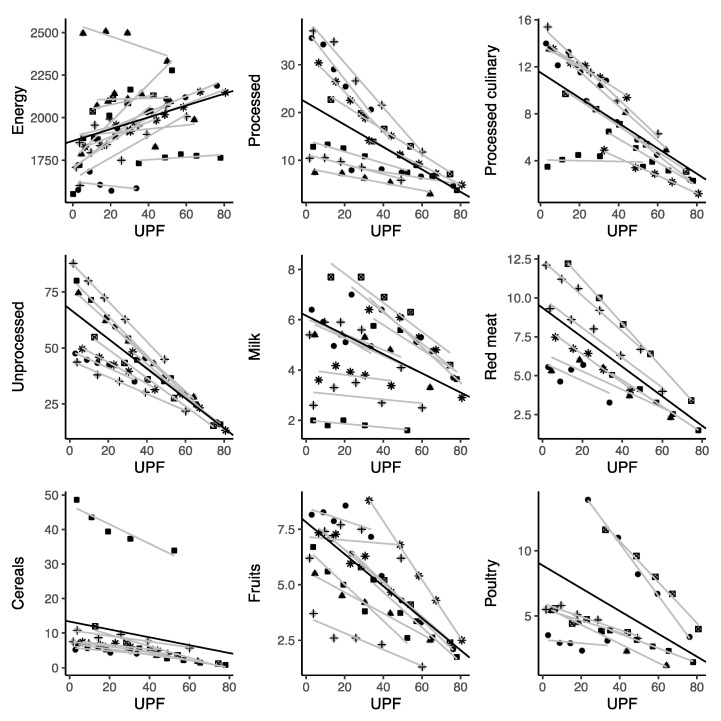

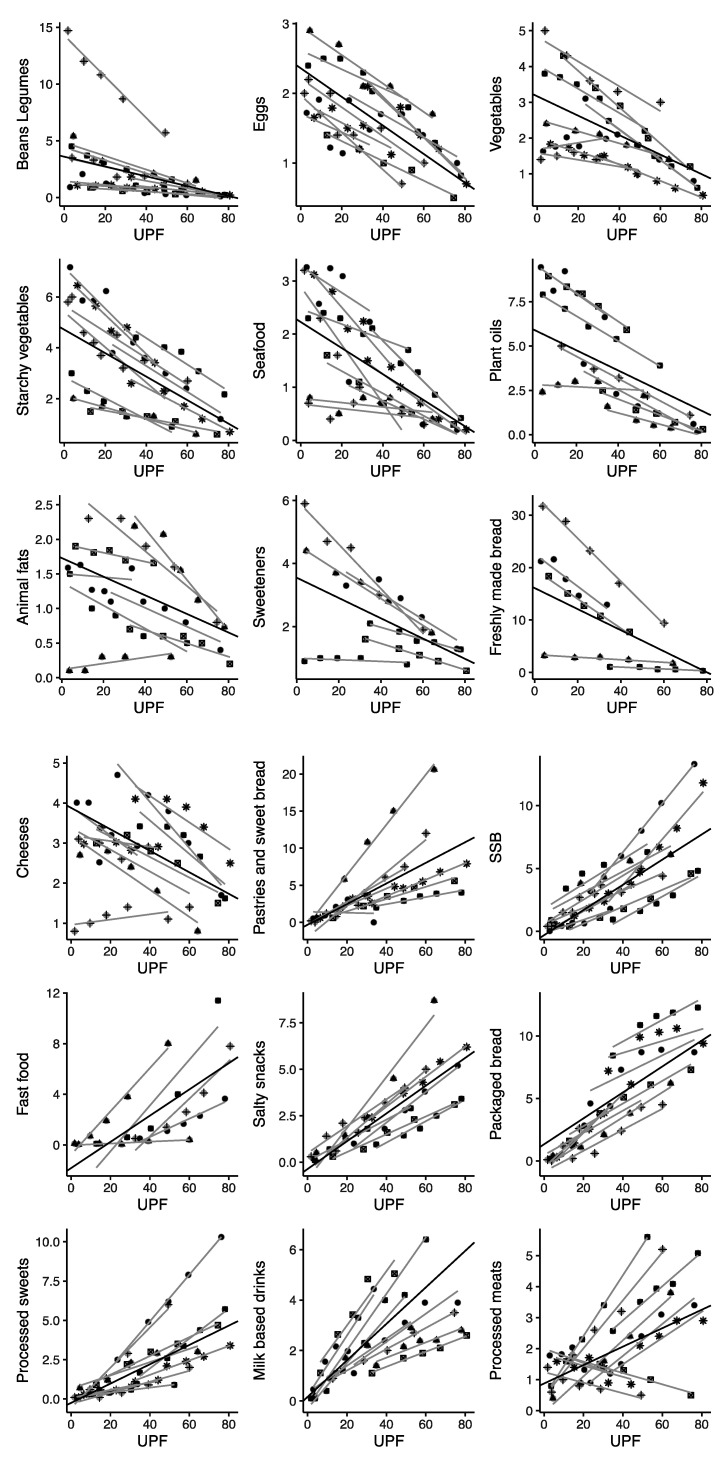

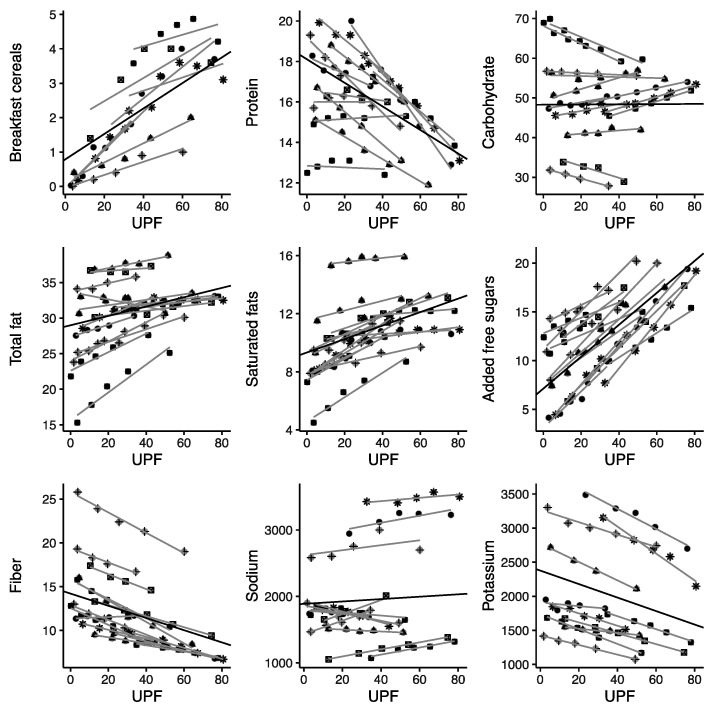
Scatter plots for the correlation between ultra-processed food consumption and selected food items in nationally representative samples. Symbols represent different cohorts; light lines represent linear regression coefficients of individual studies; bold lines represent summary estimates of the average variation in each food item for a 1% increase in ultra-processed food intake (as a percentage of daily energy).

The consumption of UPFs as %E was positively linearly correlated with total energy and added sugars for most individual UPFs (including sugar-sweetened beverages; packaged bread; processed sweets; milk-based drinks; processed meats; breakfast cereals; fast food; salty snacks; cookies, pastries, and sweet bread; and processed dairy products—despite the availability of only one related study for this category). By contrast, UPF consumption was negatively linearly correlated with all the unprocessed foods included in this analysis (total unprocessed foods, red meat, poultry, cereals, milk, fruits, starchy vegetables, vegetables, eggs, seafood, beans, and legumes), processed culinary ingredients (total processed culinary ingredients, plant oils, animal fats), and processed foods (total processed foods, cheeses) (Figure 2). Among macronutrients, UPF consumption was linearly positively associated with total fat and saturated fats, and negatively associated with protein, fiber, and certain micronutrients including potassium, magnesium, vitamin C (marginally), vitamin D, zinc, phosphorus, vitamin B12, and niacin (Figure 2).

When considering relationships between the energy provided by UPFs and various other foods, the strongest negative correlations were observed for red meat, cereals, and poultry, which had relatively higher contributions to total energy intake (nearly 8%E) for UPFs at the 15%E level, with a larger decrease (about 0.1%E) for each 1-point increase in %E from UPF intake (Table 2). Other food items consumed more frequently when minimal %E was provided by UPFs included starchy vegetables, eggs, and legumes, although their contribution to total %E was lower than that of the previously discussed group of foods (Table 2). Unhealthy trends were observed for processed culinary ingredients: a 1%E increase in the consumption of UPFs was linearly correlated with an increase in added sugars (nearly 0.2%E) and a decrease in plant oils (about 0.05%E). Among the individual representatives of UPFs, the strongest correlations were found for cookies, pastries, and sweet bread; sugar-sweetened beverages; fast food; salty snacks; processed sweets; and milk-based drinks (for each 1-point increase in the %E from UPF intake, about 7–10% was due to each of these food items) (Table 2).

UPF consumption positively correlated with total caloric intake; each additional 1%E from UPFs correlated with a 3.47 Kcal increase (95% CI: 1.47; 5.47), corresponding with a variation of about 200 Kcal daily between the 15%E from UPFs and the 75%E from the UPFs groups (Table 3). In regard to nutrient intake, increased UPF consumption inversely correlated with protein and fiber intake (about a 0.07%E decrease for each 1-point increase in %E from UPF intake) and correlated directly with total and saturated fats (about a 0.05%E increase for each 1-point increase in %E from UPF intake) (Table 3). The contribution of macronutrients to the overall diet changed considerably for protein, total fats, and fiber when comparing the 15%E from UPFs with the 75%E from UPFs groups (Table 3). The increase in UPF consumption also correlated with trends toward an inadequate dietary intake of micronutrients, with a significant reduction in potassium, magnesium, vitamin A, C, D, E, B12, niacin, zinc, and phosphorus detected (Table 3).

## 4. Discussion

In this study, we examined the existing evidence regarding the relationship between parameters of dietary nutritional quality and UPF consumption in nationally representative samples. The results indicate a significant consumption of UPFs in the countries investigated, accounting for up to almost 80% of total caloric intake in the highest UPF consumption quintiles of the US and Canada, with confectionery (categorized as “cookies, pastries, and sweet bread”) and sugar-sweetened beverages representing the most commonly consumed UPF groups. When considered in relation to other food groups, an inverse linear relation between UPFs and less processed foods was evident, suggesting that UPFs were not sporadically consumed in an isolated manner, but instead characterized entire dietary patterns and were consumed at the expense of unprocessed foods. An increased UPF intake also correlated with a substantial increase in free sugars and total and saturated fats, as well as a decrease in fiber, protein, potassium, zinc, and magnesium, and vitamins A, C, D, E, B12, and niacin. These results are not entirely surprising as the addition of ingredients such as sugars or fats are defining elements of UPFs [8]. On the other hand, the results related to micronutrients are intriguing and deserve further attention, as it has been noted that the fortification of some UPFs can help, in some contexts, to avoid specific nutritional deficiencies [36].

This quantitative evaluation is important to underline that—in addition to the mechanisms hypothesized to explain the relationship between UPFs and human health (i.e., food additives, alteration of the food matrix, etc.) [37]—the current NOVA classification system identifies as UPFs a substantial number of foods formulated with an excess of one or more nutrients (mainly sugars and/or lipids) that are consumed as alternatives to nutrient-rich foods, thus inevitably leading to a nutritionally unbalanced diet when consumed as a stable and consistent part of the diet. On one side, these results may provide an explanation for the detrimental effect of UPF consumption on markers of human health. On the other hand, it is noteworthy that these data support criticisms of UPF classification, which is theoretically based on the extent of food processing, but also reflects the varying nutritional quality of the food products within different NOVA groups. Thus, it appears important to investigate further whether the detrimental results attributed to UPFs are due to their processing or their unfavorable nutritional quality.

Based on our estimations, a diet characterized by 50%E from UPFs would obtain nearly one fourth of its daily energy from cookies, pastries, and sweet bread; packaged bread; fast foods; sugar-sweetened beverages; and processed sweets alone, while these food groups can approach half of the total daily energy intake in those countries that acquire 70–75%E from UPFs. Together with excess consumption of the previously mentioned foods, the consumption of UPFs was found to have an inverse linear relationship with the consumption of unprocessed foods; we estimated that the largest decreases in consumption associated with higher consumption of UPFs were for poultry and red meat (among animal-based foods) and cereals and fruits (among plant-based ones). For animal-based foods, the estimated variation between the low and high share of %E from UPFs roughly corresponded to a shift from one serving per day to two servings per week. Similarly, the variation in cereal and legume intake would lead to a reduction to roughly one serving per week. Considering these results on dietary protein sources, it is not surprising that the strongest negative impact of increased UPF consumption was on protein intake. Moreover, the cumulative effect of increased UPF consumption and decreased fruit and vegetable intake likely impacts the overall intake of fiber and the majority of vitamins investigated. These findings may quantitatively explain the so-called “nutrition transition” phenomenon observed across several world regions—including low- and middle-income countries—with a substantial shift from traditional to “Westernized” dietary patterns [38]. Diet quality at the global level has experienced a substantial deterioration when considering dietary components such as processed meats, sugar-sweetened beverages, saturated and trans fats, cholesterol, and sodium [39]. However, when considering the potential impact on health, diets lacking in whole grains/dietary fiber and high in sodium are considered to be the risk factors most responsible for premature mortality [40]. While these considerations are important, it is noteworthy that the higher consumption of UPFs did not significantly contribute to a higher sodium intake (as could be hypothesized).

Some studies conducted using the same samples included in the present meta-analysis also provided additional data regarding the contribution of UPFs to added sugars in diets. Pooled data from the studies demonstrated that diets with 15 to 75%E provided by UPFs resulted in an increase in the daily intake of added sugars from about 8.5 to 19%E (roughly, an extra 200 kcal per day in a 2000 kcal/day diet). Among the countries with the highest UPF consumption, nationally representative data from the US revealed that UPFs comprised 57.9% of energy intake, and contributed 89.7% of the energy intake from added sugars, with a total of 82.1% of Americans in the highest quintile exceeding the recommended limit of 10% of energy from added sugars, compared with 26.4% in the lowest [41]. Similarly, UPFs accounted for 56.8% of total energy intake and 64.7% of total free sugars in the UK diet, with 74.3% of the adults in the highest quintile of UPF consumption exceeding the recommended daily limit for free sugars, compared with 9.6% in the lowest [42]. Finally, another survey conducted in Australia indicated that UPFs accounted for 42.0% of energy intake, and free sugars represented 11.7% of the energy intake—mostly provided by UPFs—with 82.1% of the individuals in the highest quintile of UPF consumption exceeding the dietary recommendation for sugars, compared with 21.7% in the lowest quintile [43]. Several studies drew attention to the health burden associated with excessive sugar consumption (e.g., from sugar-sweetened beverages), resulting in a 10 to 20% increased risk of obesity, type 2 diabetes, and hypertension [44], and was estimated to be responsible globally for about 133,000 diabetes mellitus-related deaths, 45,000 cardiovascular disease-related deaths, and 6450 deaths associated with cancers each year [45]. These data, while alarming, provide a potential target for new food policy initiatives aiming to reduce the impact of UPFs on the nutritional quality of diets [46].

In the present study, the decision not to include data from non-national samples was made due to the enormous diversity of studies, with some being too small or including specific types of participants (e.g., students, children, older individuals, specific categories of workers, etc.), which could lead to potential selection biases and poor representativeness of a certain country. However, when compared with worldwide evaluations of UPF consumption, the presented results are reasonably consistent and comparable [47]. For instance, a study conducted on 740 Brazilian rural farmers reported that the largest caloric contribution to their diet was provided by minimally processed foods (64.7%), while UPFs contributed only minimally (5.2%); however, a higher dietary share of UPFs led to a lower intake of all macro- and micronutrients, without a distinction between healthy and unhealthy ones [48]. A study conducted on 223 Colombian school children from low- to middle-income families reported that increased shares of processed foods and UPFs in the diet (representing 34.4% of total daily energy intake) was accompanied by a lower intake of vitamins A, B12, C, and E, calcium, and zinc, and a higher intake of sodium, sugar, and trans-fatty acids [49]. Another study conducted on First Nations people living in Canada (*n* = 3700) revealed that UPFs accounted for 53.9% of their total energy intake, contributing to a higher intake of free sugars, saturated fats, sodium, calcium, and vitamin C, while protein, fiber, potassium, iron, and vitamin A intake was decreased [50,51]. A study conducted on 617 community-dwelling adults living in Japan reported that UPFs contributed 38.2% of their total daily energy intake; a linear trend was seen between the dietary share of ultra-processed foods (grouped in tertiles) and the dietary content of total and saturated fat, while an inverse relationship was observed for protein, vitamin K, vitamin B6, dietary fiber, magnesium, phosphorus, and iron [52].

In addition, data from large cohorts not meeting the criteria for inclusion in the present meta-analysis are substantially in line with those presented in our study. Findings from the Moli-Sani (a cohort of about 20,000 individuals living in a southern region of Italy) revealed significant decreasing trends in the consumption of fruit and vegetables, legumes, alcohol, and fiber, and an increasing consumption of sugar, saturated fats, and cholesterol across quintiles of UPF consumption [53]. Similar findings on fruit and vegetables, dietary fiber, and saturated fats were reported in the SUN cohort, including nearly 20,000 Spanish university graduates recruited on a voluntary basis [54]. Similarly, in the Nutrient-Santè cohort (including about 110,260 individual adult volunteers recruited in France through a web-based survey), a trend of a decreasing intake of dietary fiber and alcohol and an increasing intake of sugar and saturated fatty acids—across quartiles of UPF consumption—was observed [55]. Most micronutrients were not explored in the aforementioned cohorts, whereas other nutrients, such as sodium, did not appear to be significantly related to UPF consumption, which is in line with the results provided in this meta-analysis. By contrast, some macronutrients, such as proteins, did not show clear decreasing trends with higher consumption of UPFs; thus, further investigation on this matter is needed to confirm the findings provided in our study. Overall, our results are in reasonable agreement with those presented in nationally representative studies, especially regarding the impact of UPFs on added sugars and saturated fats; however, the impact on other nutrients may differ across studies and populations.

The results of this study should be considered in light of some limitations. First, the current analysis is limited to the currently available data. The limited number of samples from European or Asian countries strongly limit the generalizability of these results to populations living in the aforementioned areas; nevertheless, the findings seem reasonably homogeneous and consistent across countries. Thus, there is no reason to doubt that the distribution of these variables would be similar around the globe. Nonetheless, comprehensive conclusions should only be made when more samples covering a broader number of nations become available. Secondly, most of the available data relate to the dietary habits of both children and adults; while this is important in providing a comprehensive picture of the dietary consumption of UPFs across all age groups, we cannot rule out the possibility of differences existing between older and younger individuals—especially given that the generational gap represents one of the main drivers of UPF consumption.

## 5. Conclusions

In conclusion, while the share of UPFs in total daily energy intake varies across countries, there is a consistent correlation between the increased consumption of UPFs and the worsening nutritional quality of diet. It is important to better elucidate whether these results can be attributed to the level and/or type of processing, or to the unfavorable nutritional quality of UPFs. A better understanding of this matter will allow for better interpretation of the results of future studies in this field. This study underlines the importance of investigating UPFs not just as unhealthy foods consumed within the context of the diet, but as a group of foods that characterize whole diets consumed in place of healthier ones.

## Figures and Tables

**Table 1:** Main characteristics of the nationally representative samples included in the analysis.

Author, Year	Survey Name	Country	Years	Number; Sex	Age	Dietary Assessment	%E UPFs, (Mean)	Lowest Quintile%E UPFs (Mean)	Upper Quintile%E UPFs (Mean)
Shim, 2021 [34]	Korea National Health and Nutrition Examination Survey (KNHANES)	Korea	2016–2018	16,657; 49.7% females	19+ years old	24-h dietary recall	25.1	3.6	52.4
Ruggiero, 2021 [35]	Italian Nutrition & HEalth Survey (INHES)	Italy	2010–2013	9078	5–97 years old	24-h dietary recall	17.8	4.0	35.0
Costa de Miranda, 2021 [32]	National Food, Nutrition and Physical Activity Survey (IAN-AF)	Portugal	2015–2016	3852 (3102 adults, 750 elderly)	18–64 or 65+ years old	two 24-h dietary recalls	24.0	6.5	44.1
Calixto Andrade, 2021 [33]	Étude Nationale Nutrition Santé Survey (ENNS)	France	2006–2007	2642; 63.3% females	18–74 years old	three 24-h dietary recalls	31.1	12.8	51.5
Parra, 2019 [31]	National Nutrition Survey and the Demographic and Health National Survey of Colombia (ENDS)	Colombia	2004–2005	38,643; 51.9% females	2–64 years	24-h dietary recall	15.9	0.2	41.1
Marron-Ponce, 2019 [30]	Mexican National Health and Nutrition Survey	Mexico	2012	10,087; 50.5% females	1 year or older (50% adults aged 20 to 59 years)	24-h dietary recall	30.0	4.5	64.2
Machado, 2019 [29]	National Nutrition and Physical Activity Survey (NNPAS)	Australia	2011–2012	12,153	2+ years old	two 24-h dietary recalls	42.0	12.8	74.5
Cediel, 2021 [28]	National Dietary Survey in Chile (ENCA)	Chile	2010	4920	2+ years old	24-h dietary recall	28.6	3.8	60.1
Rauber, 2018 [27]	UK National Diet and Nutrition Survey (NDNS)	United Kingdom	2008–2014	9364 (4729 adults and 4635 children)	1.5 year or older	4-day diary	56.8	34.9	78.1
Chen, 2018 [26]	Nutrition and Health Surveys in Taiwan (NAHSIT)	Taiwan	1993–1996, 2011	2062	16–18 years old	24-h dietary recall	19.5	5.4	49.8
Martinez Steele, 2017 [25]	National Health and Nutrition Examination Survey (NHANES)	United States	2009–2010	9317	1+ years old	two 24-h dietary recalls	57.5	32.6	80.7
Moubarac, 2017 [24]	Canadian Community Health Survey (CCHS)	Canada	2004	33,694; 46,5% females	2+ years old (55,1% aged 2–18 years)	two 24-h dietary recalls	47.7	23.5	76.2
Costa Louzada, 2015 [22]; Louzada, 2015 [23]	Brazilian Family Budgets Survey (POF)	Brazil	2008–2009	32,898	10+ years old	two 24-h dietary records	21.5	1.8	49.2

**Table 2:** Variation in specific food item intake by contribution of ultra-processed foods to total energy consumption and slope of nationally representative samples. Slopes represent the variation in intake of a specific food item when the share of %E from UPFs increased by 1%.

Variable	Datasets (Studies)	Estimates (95% CI) in Categories of UPF Contribution				*p* for Slope
Ultra-Processed Foods (%)		15% UPF	50% UPF	75% UPF	Slope	
Sugar-sweetened beverages (%)	10 (9)	1.20 (0.44; 1.96)	4.70 (3.43; 5.97)	7.21 (5.30; 9.11)	0.10 (0.07; 0.13)	0.001
Packaged bread (%)	9 (8)	2.89 (1.27; 4.51)	6.52 (5.07; 7.96)	9.11 (7.55; 10.67)	0.10 (0.08; 0.13)	<0.001
Processed sweets (%)	10 (9)	0.67 (0.35; 1.00)	2.83 (1.64; 4.01)	4.36 (2.48; 6.24)	0.06 (0.03; 0.09)	<0.001
Milk-based drinks (%)	9 (8)	1.25 (0.90; 1.59)	3.78 (2.59; 4.97)	5.59 (3.77; 7.41)	0.07 (0.05; 0.10)	0.037
Processed meats (%)	10 (9)	1.32 (1.02; 1.63)	2.36 (1.34; 3.38)	3.11 (1.41; 4.81)	0.03 (0.00; 0.06)	<0.001
Breakfast cereals (%)	8 (7)	1.34 (0.65; 2.04)	2.64 (1.91; 3.36)	3.56 (2.69; 4.43)	0.04 (0.03; 0.05)	<0.001
Fast food (%)	5 (5)	0.00 (0.00; 1.16)	3.35 (0.67; 6.02)	5.94 (1.86; 10.01)	0.10 (0.04; 0.17)	0.001
Salty snacks (%)	8 (8)	0.75 (0.28; 1.22)	3.37 (2.48; 4.26)	5.24 (3.98; 6.50)	0.07 (0.06; 0.09)	<0.001
Cookies, pastries, and sweet bread (%)	8 (7)	1.76 (0.89; 2.63)	6.63 (3.47; 9.80)	10.12 (5.28; 14.95)	0.14 (0.07; 0.21)	<0.001
Sweeteners (%)	6 (6)	3.07 (1.86; 4.28)	1.95 (1.32; 2.57)	1.14 (0.86; 1.43)	−0.03 (−0.05; −0.01)	<0.001
Unpackaged freshly made bread (%)	5 (4)	13.08 (3.51; 22.65)	6.05 (1.73; 10.38)	1.04 (−0.42; 2.49)	−0.20 (−0.35; −0.05)	0.010
Unprocessed foods (%)	10 (9)	57.41 (49.98; 64.83)	34.12 (30.55; 37.69)	17.49 (16.27; 18.71)	−0.67 (−0.78; −0.55)	<0.001
Red meat (%)	7 (6)	7.94 (6.02; 9.86)	4.62 (3.40; 5.84)	2.25 (1.48; 3.02)	−0.09 (−0.12; −0.07)	<0.001
Poultry (%)	7 (6)	7.57 (3.71; 11.43)	4.52 (2.27; 6.76)	2.33 (1.09; 3.58)	−0.09 (−0.14; −0.04)	<0.001
Cereals (%)	8 (7)	11.65 (2.85; 20.46)	7.67 (0.65; 14.68)	4.82 (−0.98; 10.62)	−0.11 (−0.17; −0.06)	<0.001
Milk (%)	10 (9)	5.59 (4.29; 6.89)	4.26 (3.35; 5.16)	3.30 (2.65; 3.95)	−0.04 (−0.05; −0.03)	<0.001
Fruits (%)	10 (9)	6.74 (5.41; 8.07)	4.22 (3.29; 5.15)	2.42 (1.59; 3.24)	−0.07 (−0.09; −0.05)	<0.001
Starchy vegetables (%)	10 (9)	4.02 (2.97; 5.08)	2.41 (1.72; 3.10)	1.25 (0.76; 1.75)	−0.05 (−0.06; −0.03)	<0.001
Vegetables (%)	10 (9)	2.76 (2.09; 3.43)	1.81 (1.41; 2.21)	1.13 (0.77; 1.49)	−0.03 (−0.04; −0.02)	<0.001
Eggs (%)	10 (9)	2.05 (1.74; 2.37)	1.33 (1.05; 1.62)	0.82 (0.52; 1.12)	−0.02 (−0.02; −0.02)	<0.001
Seafood (%)	10 (9)	1.86 (1.34; 2.38)	1.00 (0.69; 1.32)	0.39 (0.07; 0.71)	−0.02 (−0.03; −0.02)	<0.001
Beans and legumes (%)	10 (9)	2.95 (0.95; 4.94)	1.43 (0.52; 2.35)	0.35 (0.19; 0.51)	−0.04 (−0.07; −0.01)	0.006
Processed culinary ingredients (%)	9 (8)	9.90 (7.81; 11.98)	6.13 (4.85; 7.40)	3.44 (2.48; 4.39)	−0.11 (−0.14; −0.08)	<0.001
Added free sugars (%)	14 (12)	9.58 (7.61; 11.56)	15.31 (13.85; 16.78)	19.41 (17.94; 20.87)	0.16 (0.13; 0.19)	<0.001
Plant oils (%)	8 (7)	5.04 (3.28; 6.81)	3.06 (1.77; 4.35)	1.64 (0.65; 2.64)	−0.06 (−0.07; −0.04)	<0.001
Animal fats (%)	8 (7)	1.53 (0.92; 2.14)	1.06 (0.67; 1.44)	0.72 (0.41; 1.04)	−0.01 (−0.02; 0.00)	0.002
Processed foods (%)	10 (9)	18.60 (13.01; 24.19)	10.39 (7.92; 12.86)	4.52 (3.37; 5.67)	−0.23 (−0.33; −0.14)	<0.001
Cheeses (%)	9 (8)	3.48 (2.59; 4.37)	2.54 (1.96; 3.11)	1.87 (1.37; 2.37)	−0.03 (−0.04; −0.01)	<0.001

**Table 3:** Variation in specific nutrient intake by contribution of ultra-processed foods to total energy consumption and slope of nationally representative samples. Slopes represent the variation in intake of a specific food item when the share of %E from UPFs increased by 1%.

Variable	Datasets (Studies)	Estimates (95% CI) in Categories of UPF Contribution				*p* for Slope
Ultra-Processed Foods (%)		15% UPF	50% UPF	75% UPF	Slope	
Energy (kcal)	14 (12)	1915.25 (1804.35; 2026.15)	2036.70 (1934.50; 2138.90)	2123.45 (2000.51; 2246.38)	3.47 (1.47; 5.47)	<0.001
**Nutrients**						
Protein (%)	13 (11)	17.23 (15.95; 18.51)	15.19 (14.38; 16.01)	13.74 (12.84; 14.64)	−0.06 (−0.08; −0.03)	<0.001
Carbohydrate (%)	13 (11)	48.29 (42.46; 54.12)	48.37 (42.65; 54.09)	48.43 (42.12; 54.74)	0.00 (−0.07; 0.08)	0.949
Total fat (%)	15 (13)	29.97 (27.58; 32.36)	32.27 (30.55; 33.98)	33.91 (32.41; 35.41)	0.07 (0.04; 0.10)	<0.001
Saturated fats (%)	15 (13)	10.03 (8.94; 11.11)	11.65 (10.79; 12.50)	12.81 (11.95; 13.66)	0.05 (0.03; 0.06)	<0.001
Trans fats (%)	3 (3)	0.70 (0.08; 1.33)	0.96 (−0.05; 1.98)	1.14 (−0.15; 2.44)	0.01 (0.00; 0.02)	0.211
Fiber (g/1000 kcal)	14 (12)	13.16 (11.00; 15.33)	10.73 (8.89; 12.57)	8.99 (7.26; 10.72)	−0.07 (−0.09; −0.05)	<0.001
**Micronutrients**						
Sodium (mg/1000 kcal)	12 (10)	1914.70 (1504.16; 2325.23)	1977.65 (1551.45; 2403.84)	2022.61 (1566.01; 2479.21)	1.80 (−1.62; 5.21)	0.302
Potassium (mg/1000 kcal)	11 (10)	2228.24 (1735.90; 2720.58)	1881.86 (1456.07; 2307.65)	1634.45 (1248.90; 2019.99)	−9.90 (−12.60; −7.19)	<0.001
Iron (mg/1000 kcal)	4 (4)	10.09 (4.22; 15.95)	9.04 (4.42; 13.66)	8.30 (4.53; 12.06)	−0.03 (−0.07; 0.01)	0.120
Magnesium (mg/1000 kcal)	4 (4)	200.63 (141.17; 260.09)	161.53 (113.10; 209.96)	133.60 (92.75; 174.46)	−1.12 (−1.46; −0.78)	<0.001
Calcium (mg/1000 kcal)	5 (5)	433.84 (299.41; 568.26)	401.01 (299.50; 502.52)	377.57 (294.55; 460.58)	−0.94 (−2.13; 0.26)	0.123
Vitamin A (μg/1000 kcal)	5 (5)	431.11 (232.39; 629.83)	332.65 (221.80; 443.50)	262.32 (203.20; 321.43)	−2.81 (−5.48; −0.15)	0.038
Vitamin C (mg/1000 kcal)	5 (5)	79.17 (47.78; 110.57)	66.79 (39.07; 94.52)	57.95 (32.05; 83.85)	−0.35 (−0.55; −0.16)	<0.001
Vitamin D (μg/1000 kcal)	4 (4)	3.73 (2.25; 5.21)	2.81 (1.75; 3.86)	2.14 (1.39; 2.90)	−0.03 (−0.04; −0.01)	<0.001
Zinc (mg/1000 kcal)	3 (3)	6.60 (6.12; 7.08)	5.46 (5.01; 5.91)	4.64 (4.10; 5.19)	−0.03 (−0.04; −0.02)	<0.001
Phosphorus (mg/1000 kcal)	4 (4)	666.55 (527.35; 805.76)	582.91 (475.76; 690.07)	523.17 (436.17; 610.17)	−2.39 (−3.46; −1.32)	<0.001
Vitamin E (mg/1000 kcal)	3 (3)	5.41 (1.12; 9.69)	4.68 (0.99; 8.37)	4.16 (0.89; 7.43)	−0.02 (−0.04; 0.00)	0.016
Vitamin B12 (μg/1000 kcal)	3 (3)	3.78 (2.10; 5.45)	2.95 (1.33; 4.57)	2.36 (0.77; 3.95)	−0.02 (−0.03; −0.02)	<0.001
Thiamin (mg/1000 kcal)	3 (3)	1.06 (0.49; 1.63)	0.93 (0.65; 1.21)	0.84 (0.76; 0.93)	0.00 (−0.01; 0.00)	0.406
Riboflavin (mg/1000 kcal)	3 (3)	1.18 (0.84; 1.53)	1.07 (0.79; 1.35)	0.99 (0.72; 1.26)	0.00 (−0.01; 0.00)	0.074
Niacin (mg/1000 kcal)	4 (4)	16.97 (9.26; 24.68)	14.18 (8.38; 19.99)	12.19 (7.72; 16.65)	−0.08 (−0.14; −0.02)	0.005

## Supplementary Materials

The following are available online at https://www.mdpi.com/article/10.3390/nu13103390/s1, Table S1: PRISMA checklist.
